# A clock for all seasons

**DOI:** 10.1007/s00359-024-01711-8

**Published:** 2024-06-19

**Authors:** Charlotte Helfrich-Förster, Dirk Rieger

**Affiliations:** Neurobiology and Genetics, Biocentre, University of Würzburg, Würzburg, Germany

**Keywords:** Circadian clock, Photoperiodism, Dual oscillator model, Colin S. Pittendrigh, Erwin Bünning, David S. Saunders

## Abstract

Circadian clocks play an essential role in adapting locomotor activity as well as physiological, and metabolic rhythms of organisms to the day-night cycles on Earth during the four seasons. In addition, they can serve as a time reference for measuring day length and adapt organisms in advance to annual changes in the environment, which can be particularly pronounced at higher latitudes. The physiological responses of organisms to day length are also known as photoperiodism. This special issue of the Journal of Comparative Physiology A aims to account for diurnal and photoperiodic adaptations by presenting a collection of ten review articles, five original research articles, and three perspective pieces. The contributions include historical accounts, circadian and photoperiodic clock models, epigenetic, molecular, and neuronal mechanisms of seasonal adaptations, latitudinal differences in photoperiodic responses and studies in the wild that address the challenges of global change.

## Introduction

The evolution of living beings occurred in a cyclical environment in which numerous factors, including light, temperature, humidity, and food availability, oscillated on a daily 24-hour rhythm. Endogenous circadian clocks assist organisms in anticipating and adjusting to these fluctuations. Furthermore, they enable individual animals to measure time, which is important for a time memory (Beer et al. 2024), and they help animal populations to synchronize crucial steps in their life (such as mating or eclosing from the pupal case in insects) at the optimal time of the day (Beer and Helfrich-Förster 2020). They also provide an internal time reference for birds and insects that orient themselves via a sun compass, which is necessary to compensate for the sun’s predictable daily motion (Kramer 1952; Mouritsen and Frost 2002). In addition to all these functions, circadian clocks are believed to serve as an internal reference for measuring day length (Bünning 1936). The change in day length is a reliable indicator of the upcoming season, and it is very important for all organisms to prepare for it in advance. Typical photoperiodic responses to increasing day length in spring are increased growth and reproduction, while decreasing day length leads to hibernation (diapause in insects) or migration to warmer regions. A failure to adapt in time to winter will ultimately result in death, while a failure to adapt in time to spring/summer will result in no or very few offspring. Bünning (1932) therefore concluded that the possession of a circadian clock for measuring day length represents a considerable selection advantage.

It is now almost 50 years since Colin S. Pittendrigh and Serge Daan published their influential article “A functional analysis of circadian pacemakers in rodents. V. Pacemaker structure: a clock for all seasons” (Pittendrigh and Daan 1976c), as the last in a series of five articles published in one issue of the Journal of Comparative Physiology A (JCP A). These 5 research papers are among the most cited in JCP A (3714 citations in total), having made the issue in which these articles appeared the “Bible of Chronobiology” used by hundreds of students and scientists (Zupanc et al. 2024). In their work, Pittendrigh and Daan describe the basic properties of circadian clocks (here referred to as circadian pacemakers) and explore how the entrainment of the circadian clock to the 24-hour day adapts animals to different environmental cues including the annual variations in day length (Daan and Pittendrigh 1976a, b; Pittendrigh and Daan 1976a; Daan and Pittendrigh 1976b; c). They propose that the clock consists of two separate oscillators with different responses to light: one oscillator accelerates and the other decelerates upon light exposure. Under light–dark cycles, the first oscillator tracks dawn and is therefore called the morning (M) oscillator, while the second tracks dusk and is called the evening (E) oscillator. Due to the different properties of the M and E oscillators, the two activity bouts in the morning and evening are close together under short days and far apart under long days. Thus, the dual oscillator model proposes that M and E activity bouts change their phase angle, leading to different waveforms of the activity rhythm at different photoperiods. In summer, morning and evening activity of diurnal animals are far apart, allowing the animals to rest during the heat around midday. In contrast, the two peaks are close together on cold winter days, so that the animals can be active during the warmer part of the day. Furthermore, the time difference between the two oscillators serves as an indicator of day length, which can be employed to anticipate the season and prepare for hibernation if it falls below a specified threshold in the fall, or to prepare for breeding if it rises above a certain threshold in the spring.

This model of day length measurement is referred to as the “Internal Coincidence” model, as it measures the time between two internal oscillators. It was first proposed for the circadian clock of the jewel wasp, *Nasonia vitripennis*, by David S. Saunders (Saunders 1974). The Internal Coincidence Model contrasts with the Bünning’s (1936) External Coincidence Model, which is based on observations of flowering in the bean *Phaseolus multiflorus*. The External Coincidence Model proposes that a single oscillator (or a population of oscillators) possesses a particular light-sensitive or photoinducible phase. If light falls into this phase, the photoperiodic response is induced. In this instance, the concurrence between the oscillator and the external light-dark cycle serves as a cue for the photoperiodic response (Pittendrigh and Minis 1964; Saunders 1978).

Despite the overwhelming evidence that circadian clocks are important internal time references for the measurement of day length in several organisms, there is also evidence that day length measurement can function without the contribution of circadian clocks. The “hourglass model”, originally developed for aphids, predicts that a hypothetical substance accumulates during darkness and once this substance reaches a certain threshold, the photoperiodic response is triggered (Lees 1973). This model is independent of the circadian clock. However, as shown by Saunders and Lewis (1987) in a theoretical model, there can be a smooth transition between external coincidence and the hourglass, suggesting that anything in between is possible in living systems. Indeed, recent studies show that different species have evolved different ways to measure day length and to adapt their physiology and metabolism to the seasons, and one possibility is the use of weakly damped clocks that are highly plastic (Bertolini et al. 2019; Lankinen et al. 2021).

Over the past two decades, significant progress has been made in elucidating the genetic, molecular, and neuronal mechanisms underlying seasonal adaptation (Wood and Loudon 2014; Takeda and Suzuki 2022). Moreover, after decades of research conducted under laboratory conditions, the “wild clocks” approach has begun, which aims to study wild species in their natural habitats. This approach brings together circadian biology and ecology (Kronfeld-Schor et al. 2013; Schwartz et al. 2017). Such studies are suitable for elucidating the consequences of global change on the interplay between circadian and circannual clocks. The articles in this special issue review and highlight research on seasonal adaptation, circadian clocks, and the role of these clocks in measuring day length. This special issue is dedicated to David S. Saunders (1935–2023) and Wolfgang Engelmann (1934–2023), who can be regarded as pioneers in photoperiodism and circadian clock research. This special issue contains the final article by David S. Saunders, which he completed shortly before his passing.

### Contributions to this special issue

This special issue comprises ten review articles, five original research papers and three perspective pieces, which collectively address a diverse set of questions pertaining to daily and seasonal timing in invertebrates (primarily insects), birds, and mammals. The articles are loosely related to specific topics, but most of them cover additional areas or have implications for other aspects of timekeeping. In the following section, we will summarize the main points and conclusions presented by the authors of the various contributions and contextualize them within the broader framework of related findings.

### Historical perspective

Bünning (1936) proposed that the endogenous clock is used to measure day length in order to initiate photoperiodic responses. His seminal publication initiated years of contentious debate until it ultimately became the foundational axiom of rhythm research and the theoretical foundation of chronobiology. Charlotte Helfrich-Förster provides an overview of the initial experiments conducted by Bünning and his PhD student Wolfgang Engelmann, which are primarily published in German and therefore challenging for non-native speakers to access (Helfrich-Förster 2024). In his doctoral dissertation, Engelmann conducted a pivotal experiment showing that red light pulses did not alter the circadian rhythm of the Flaming Katy, *Kalanchoë blossfeldiana*, but inhibited its photoperiodic flowering when applied during the scotophilic phase of the plant. These experiments provide the foundation for our contemporary understanding of photoperiodic timing in plants, but they can also be extended to animals. Most importantly, Engelmann’s study demonstrated the extraordinary light sensitivity of the photoperiodic system, which led Bünning to hypothesize that some plants lower their leaves during the night to prevent moonlight from interfering with the measurement of daylength and flower induction (Bünning and Moser 1969). This brings to mind the disturbing effects of artificial light at night on the timing system of European hamsters, different bird species, and other seasonal animals, as discussed by Stefanie Monecke, and by Barbara Helm and Miriam Liedvogel in the topic “Clocks in the Wild and Effects of Global Changes on Seasonal Responses” (Monecke 2024; Helm and Liedvogel 2024).

The second article in this topic provides an overview of the life and scientific work of David S. Saunders. Saunders made significant contributions to our understanding of insect reproduction and adaptation to seasonal changes on our planet (Helfrich-Förster 2023). Most importantly, he was a pioneer in demonstrating the role of the circadian clock in insect photoperiodic time measurement, first in the jewel wasp *Nasonia vitripennis*, and subsequently in a number of species of flies. He has published numerous scientific articles, the most recent in this special issue.

### Circadian and photoperiodic clock models

Models are essential for establishing a theoretical framework within a specific field of research. In their perspective, Jennifer A. Evans and William J. Schwarz review the origin and evolution of the dual oscillator model proposed by Pittendrigh and Daan (1976c). This model has stimulated laboratories around the world in searches to identify and localize such hypothesized M and E oscillators, or sets of oscillators, in insects, rodents, and humans (see Evans and Schwartz in this issue). In their perspective article, Evans and Schwartz (2023) recount the conceptual origin and subsequent evolution of the dual oscillator model for the circadian clock in the mammalian suprachiasmatic nucleus (SCN). They also discuss how, Pittendrigh’s and Daan’s binary conception has remained influential in our clock models and metaphors, despite our increasingly sophisticated view of this multicellular pacemaker. Their perspective encompasses both the past and the future. In addition to discussing the model, the authors address the neuronal organization of the SCN and its increasing complexity.

David S. Saunders presents an original paper on fundamental experiments on oscillator entrainment and the External and Internal Coincidence Models in insects. The conclusions presented in this paper are primarily drawn from experiments on flesh flies (*Sarcophaga* spp.) and the parasitic wasp, *Nasonia vitripennis* (Saunders 2023). As previously mentioned in the introduction, the two models serve as the theoretical foundation for the majority of photoperiodic studies. Indeed, several of the investigations included in this special issue make reference to at least one of the two models (e.g., Bradshaw et al. 2023; Colizzi et al. 2023; Floessner et al. 2023; Helfrich-Förster 2024; Hidalgo and Chiu 2023; Michel and Kervezee 2023; Vaze et al. 2023).

The fruit fly, *Drosophila melanogaster* represents the first animal in which the anatomical substrates of the M and E oscillators have been elucidated (reviewed by Yoshii et al. 2012). Taishi Yoshii and colleagues have now reconsidered the dual-oscillator model, integrating the latest findings on the fruit fly, and conclude that it is too simple to explain all findings (Yoshii et al. 2023). In their review, they propose a four-oscillator model that controls the bimodal activity rhythms. The four oscillators (two activity and two sleep oscillators) reside in different clock neurons and regulate activity in the morning and evening and sleep during the midday and at night. Bimodal rhythms are formed by interactions among the four oscillators, which may provide a rationale for the flexible waveform of activity rhythms under different photoperiod conditions. Although still hypothetical, this model offers a novel perspective on the seasonal adaptation of the two activity peaks.

In their search for the clock model that initiates diapause in the high-latitude fly *Drosophila ezoana*, Koustubh Vaze and colleagues (Vaze et al. 2023) measured the timing of M and E activity peaks at Zeitgeber cycles with different period lengths and different photoperiods and compared the results with diapause induction measured under the same conditions in a previous study (Vaze et al. 2016). The researchers discovered that this fly species employs both the morning and evening oscillators to regulate its rhythmic activity in response to varying day lengths. However, only the morning oscillator is utilized to assess night length in photoperiodic responses. Consequently, the flies utilize external coincidence to measure day length (or better night length), which is consistent with the findings of Saunders (2023) on other fly species. The authors discuss the clock protein TIMELESS as the molecular component involved in measuring night length, which aligns with the results observed in *D. melanogaster* (Abrieux et al. 2020) and *Chymomyza costata* (Stehlik et al. 2008).

## A theoretical approach

In his review, Christoph Schmal (2023) offers a theoretical perspective on photoperiodic entrainment and encoding. The author employs the mammalian SCN as a model to demonstrate that properties of the circadian system are photoperiod dependent, and that the phase of entrainment varies systematically with season. The conceptual phase oscillator models are presented at a high level of abstraction, but the intuitive interpretation of the underlying parameters allows for a straightforward analysis of the principles governing entrainment properties. The results of this class of models are presented and discussed in the context of more complex conceptual amplitude-phase oscillators, as well as contextual molecular models that take into account organism-, tissue-, and cell type-specific details.

### Genetic and molecular mechanisms of adaptation to different seasons

The field of epigenetics plays a pivotal role in the capacity of organisms to adapt to their external environment. In their review, Bettina Fishman and Eran Tauber examine recent studies on epigenetic mechanisms that are implicated in seasonal adaptation in animals (Fishman and Tauber 2023). The review is divided into three main sections, each focusing on a different epigenetic mechanism. The epigenetic mechanisms under consideration are DNA methylation, histone modifications, and non-coding RNA. Furthermore, the review examines the current understanding of how these epigenetic factors contribute to the regulation of circadian and seasonal cycles. The authors conclude that epigenetic modifications play an important role in seasonal timing, with numerous different mechanisms present in different organisms and even within individuals. The precise molecular circuitry of the photoperiodic clock remains elusive. However, studying the epigenetic regulation of seasonal timing may provide new insights into this complex biological process.

Multiple pieces of evidence suggest the involvement of circadian clock genes, such as the *timeless* gene in photoperiodic control. However, their role might be independent of their well-established role in the daily oscillation of the circadian clock. Moreover, photoperiodic control of reproductive diapause has mainly been studied in females while the circadian clock has been investigated in males. Given the idiosyncrasies of male and female physiology, Magdalena Maria Kaniewska and colleagues studied male reproductive diapause in the strongly photoperiodic linden bug *Pyrrhocoris apterus* (Kaniewska et al. 2023). They demonstrate that the males’ mating capacity is strongly dependent on photoperiod and is very low under short days. Furthermore, mutants in the genes coding for the Pigment Dispersing Factor (PDF) and Cryptochrome (CRY) are reproductively active even in short photoperiods. This suggests that these genes are involved in measuring day length in males and females. The findings presented by the authors provide further evidence of the involvement of circadian clock genes in photoperiodic timing in insects.

In *Drosophila melanogaster*, the circadian clock gene, *timeless* appears to be involved in photoperiodic adjustments to the seasons, yet our understanding of this process is far from complete. In contrast to strongly photoperiodic animals, fruit flies don’t enter a true diapause but rather enter a state of reproductive dormancy when winter sets in. For this process to occur, it is not sufficient for the day length to be shortened; a drop in temperature is also necessary. The role of temperature as a key factor in complementing the photoperiodic response remains poorly understood. Sergio Hidalgo and Joanna C. Chiu review our current understanding of fruit fly seasonal adaptation to changes in temperature and photoperiod at the molecular level, including changes in gene expression and alternative splicing of circadian clock genes (Hidalgo and Chiu 2023). In addition, they provide an overview of the neuronal and neurohormonal circuitry involved in these adaptations. As with the findings of Kaniewska et al. (2023), the study demonstrates that the neuropeptide PDF plays a role in the transfer of day length information to the neurohormonal centers that regulate reproductive dormancy.

### Neuronal mechanisms of adaptation to different seasons in different animals

In a comprehensive review, Yoshitaka Hamanaka and colleagues examine the potential neuronal and physiological mechanisms underlying circadian clock-based photoperiodic responses in insects and snails (Hamanaka et al. 2023). The authors commence with a fundamental description of the photoperiodic system, encompassing the photoperiodic clock or timer, the photoperiodic counter, the neurosecretory system, and the endocrine organs. They then evaluate the neuronal connections from the circadian clock neurons to the photoperiodic system, as far as is currently known. Finally, they provide an update on recent physiological findings, first for insects and then for snails. Although the precise mechanisms of photoperiodic responses are less clear in snails, in both animal groups, neurosecretory cells in the brain produce neurohormones that are released into the “blood” and promote egg laying under long days. Conversely, their electrical excitability is attenuated under short and medium days, which reduces oviposition. In insects, particularly the bean bug, *Riptortus pedestris*, the neuronal pathway from the clock neurons is partially elucidated. As is the case with the linden bug and other insects, the neuropeptide PDF appears to be involved. In addition, glutamate appears to play a significant role in inhibiting the neurosecretory cells that promote egg laying.

As with the linden and bean bug, the pea aphid, *Acyrthosiphon pisum*, is a paradigmatic photoperiodic species that exhibits a remarkable annual life cycle, which is tightly coupled to the seasonal changes in day length. During the spring and summer months, when days are longer, aphid populations consist exclusively of viviparous females that reproduce parthenogenetically. As autumn approaches and the days become shorter, aphids undergo a reproductive shift, generating males and oviparous sexual females, which mate and produce cold-resistant eggs that overwinter and survive the unfavourable season. While these photoperiodic responses have been well described, the nature of the timing mechanisms underlying day length discrimination remains poorly understood. In the 1960s, experiments indicated that aphids utilize an “hourglass” clock to measure the duration of the dark night. This biochemical clock accumulates a factor that reaches a critical threshold at a specific night length, thereby triggering the switch in reproductive mode (e.g. Lees 1973). Nevertheless, the photoperiodic responses of aphids can also be attributed to a strongly dampened circadian clock. Recent studies have identified the molecular components and the location of the circadian clock in the brain of the pea aphid and demonstrated that it is intricately connected to the neurohormonal system controlling aphid reproduction (Colizzi et al. 2023a; Cuti et al. 2021). In their study, Francesca S. Colizzi and colleagues provide an overview of the putative mechanisms of photoperiodic control in aphids, from the photoreceptors involved in this process to the circadian clock and the neuroendocrine system (Colizzi et al. 2023b). Once more, the neuropeptide PDF may be implicated in the interconnection between circadian clock neurons and the photoperiodic system.

In their perspective article, Stephan Michel and Laura Kervezee (2023) pose the thought-provoking question: “One seasonal clock fits all?” First, the authors review the common principles and mechanisms in photoperiodic regulation of all animals, including humans, and conclude that there are striking similarities across all species regarding the contribution of the circadian system, usage of signalling molecules from the circadian clock to the neuroendocrine system, and participation of the hormone melatonin. The authors persuasively argue that humans are also responsive to the annual changes in seasons. They find it extremely unlikely that all animals have the same seasonal clock and strongly recommend unravelling the machinery of seasonal adaptation in a multitude of organisms, including human beings.

### Latitudinal differences in photoperiodic responses

The photoperiodic responses of the same species living at different latitudes must adapt to the relevant environment in such a way that populations from higher latitudes enter diapause earlier (at still longer day length) than populations from lower latitudes. This phenomenon has been well documented in the pitcher-plant mosquito, *Wyeomyia smithii*, which has evolved from south to north and from low to high elevations in eastern North America. Here, William E. Bradshaw and colleagues present new experiments that demonstrate an increase in critical photoperiod along the seasonal gradient, while apparent involvement of the circadian clock declined in concert with the evolutionary divergence of populations (Bradshaw et al. 2023). This finding makes it unlikely that the circadian clock plays a role in the photoperiodic control of pitcher-plant mosquitoes, unless one considers that weak circadian clocks are well suited for indicating day-length, as was hypothesized for high-latitude flies (Vaze et al. 2023). In any case, the microevolutionary processes revealed within and among populations of *W. smithii* illustrate a gateway to the macroevolutionary divergence of biological timing among species and higher taxa in general.

If the circadian clock is involved in photoperiodic control, one would anticipate differences in its properties between lines of the same species that originate from different latitudes. To address this important question, Theresa S.E. Floessner and colleagues (Floessner et al. 2023) investigated two lines of jewel wasps, *Nasonia vitripennis*, originating from southern and northern latitudes, respectively. The researchers tested the induction of diapause in a range of Zeitgeber cycles with different period lengths and different photoperiods and found that diapause induction occurred in short photoperiods in all Zeitgeber cycles in the northern line. However, in the southern line, diapause only occurred in Zeitgeber cycles with periods close to 24 h. This was accompanied by a lower light sensitivity of the circadian clock in the southern line, which resulted in a wider distribution of phase angles of entrainment at a specific Zeitgeber cycle duration. In contrast, the range of entrainment decreased. The authors conclude that the circadian clock plays a role in the timing of diapause induction and propose a new model of external coincidence involving a single oscillator with a light-sensitive phase that drives the annual timing of diapause in *N. vitripennis*.

### Clocks in the wild and effects of climate change on seasonal responses

After decades of experiments conducted under controlled laboratory conditions, it is time to extend this to natural environments. In their natural habitats, animals are not continuously exposed to the light conditions provided by the experimenters; instead, they are able to hide from the light. This phenomenon is particularly evident in species living in subterranean habitats. Gisele A. Oda and Veronica S. Valentinuzzi (2023) review experiments conducted with subterranean rodents (*Ctenomys* sp.) in the laboratory and in the field under an extreme pattern of natural daily light exposure, as well as modelling results. They confirm the majority of the features of Pittendrigh and Daan’s models, while also emphasizing the necessity of integrating these models with ecophysiological techniques, methodologies, and theories to gain a comprehensive understanding of the circadian rhythm in its natural habitat. This integration is essential to fully establish the importance of the temporal dimension in ecological studies and to address relevant questions such as the role of the clock in all seasons on a changing planet.

In the contemporary era, animals are confronted with pervasive global alterations that can influence their natural temporal patterns. This is particularly evident in seasonal long-distance migratory species such as birds, which must return to their breeding sites in spring without knowledge of the weather conditions there. If they arrive too late, they will be unable to compete with species that did not migrate. In their comprehensive review, Barbara Helm and Miriam Liedvogel (2024) examine the timing mechanisms and their adaptations to a changing world in different bird species. As a collective, migratory birds are not adequately adapting to the changes. However, some species are demonstrating remarkable adjustments at the behavioural and genetic levels. To gain a comprehensive understanding of the range of responses of migratory birds to environmental change, and more broadly, the functioning of timing programs under natural conditions, it is essential to implement integrated research programs and interdisciplinary collaborations.

Global warming represents a challenge for species that are unable to migrate to higher latitudes due to limitations in their tolerance of long days. This problem affects European hamsters (*Cricetus cricetus)*, which are listed as critically endangered on the International Union for Conservation of Nature (IUCN) Red List. The temporal structure of the environment, as influenced by climate change and light pollution, may be a contributing factor to the global decline of this species. In her perspective, Monecke (2024) establishes a connection between the classical entrainment concept and conservation concepts. Monecke demonstrates that chronobiological concepts, such as the Internal Coincidence Model, can assist in elucidating the causes of the decline and may potentially support species conservation. An understanding of the species’ physiological limitations and its adaptation capacities can potentially prevent extinction at a time when classical conservation concepts have reached their limits.

### Perspectives

The aim of this special issue is to provide an insight into the topic of seasonal adaptation of organisms, from conceptual and theoretical models to possible molecular and neuronal mechanisms that control appropriate behaviour in a changing world. The species studied are not chosen at random. Some can be considered genetic model organisms that could help uncover underlying molecular mechanisms; others are genetically elusive but show unique seasonal adaptations and some can be studied in the wild. The question that runs through all the articles is: How do endogenous clocks contribute to seasonal adaptations? There is no single answer to this question. Despite fundamental similarities, the strategies are so diverse that the next generation of researchers will not run out of questions. It is to be hoped that in a drastically changing environment there will be enough time to gain these insights and, in the best case, contribute to the preservation of the diversity of organisms.

## Data Availability

No datasets were generated or analysed during the current study.

## References

[CR1] Abrieux A, Xue Y, Cai Y (2020). EYES ABSENT and TIMELESS integrate photoperiodic and temperature cues to regulate seasonal physiology in *Drosophila*. Proc Natl Acad Sci USA.

[CR2] Beer K, Helfrich-Förster C (2020). Model and non-model insects in chronobiology. Front Behav Neurosci.

[CR3] Beer K, Zupanc GKH, Helfrich-Förster C (2024). Ingeborg Beling and the time memory in honeybees. J Comp Physiol A.

[CR4] Bertolini E, Schubert FK, Zanini D (2019). Life at high latitudes does not require circadian behavioral rhythmicity under constant darkness. Curr Biol.

[CR5] Bradshaw WE, Fletcher MC, Holzapfel CM (2023) Clock–talk: have we forgotten about geographic variation? J Comp Physiol A 210. 10.1007/s00359-023-01643-9 10.1007/s00359-023-01643-9 PMC11226528 37322375

[CR6] Bünning E (1932). Über die Erblichkeit Der Tagesperiodizität bei den Phaseolus-Blättern. Jb Wiss Bot.

[CR7] Bünning E (1936). Die endonome Tagesrhythmik als Grundlage Der Photoperiodischen Reaktion. Ber Deutsch Bot Ges.

[CR8] Bünning E, Moser I (1969). Interference of moonlight with the photoperiodic measurement of time by plants, and their adaptive reaction. Proc Natl Acad Sci U S A.

[CR10] Colizzi S, Martinez-Torres, Helfrich-Förster C (2023b) The circadian and photoperiodic clock of the pea aphid. J Comp Physiol A 210. 10.1007/s00359-023-01660-8 10.1007/s00359-023-01660-8 PMC11226554 37482577

[CR9] Colizzi FS, Veenstra JA, Rezende GL, Helfrich-Förster C, Martinez-Torres D (2023). Pigment-dispersing factor is present in circadian clock neurons of pea aphids and may mediate photoperiodic signalling to insulin-producing cells. Open Biol.

[CR11] Cuti P, Barberà M, Veenstra JA, Martínez-Torres D (2021). Progress in the characterization of insulin-like peptides in aphids: immunohistochemical mapping of ILP4. Insect Biochem Mol Biol.

[CR12] Daan S, Pittendrigh CS (1976). A functional analysis of circadian pacemakers in nocturnal rodents. II. The variability of phase response curves. J Comp Physiol A.

[CR13] Daan S, Pittendrigh CS (1976). A functional analysis of circadian pacemakers in nocturnal rodents. III. Heavy water and constant light: homeostasis of frequency?. J Comp Physiol A.

[CR14] Evans JA, Schwartz WJ (2023) On the origin and evolution of the dual oscillator model underlying the photoperiodic clockwork in the suprachiasmatic nucleus. J Comp Physiol A 2010. 10.1007/s00359-023-01659-1 10.1007/s00359-023-01659-1 PMC10924288 37481773

[CR46] Fishman B, Tauber E (2023) Epigenetics and seasonal timing in animals: a concise review. J Comp Physiol A 210. https//doi.org/10.1007/s00359-023-01673-3 10.1007/s00359-023-01673-3 PMC11226475 37695537

[CR15] Floessner TSE, Dalla Benetta E, Beersma DGM, Hut RA (2023) Variation in photoperiod response corresponds to differences in circadian light sensitivity in northern and southern *Nasonia vitripennis* lines. J Comp Physiol A 2010. 10.1007/s00359-023-01674-2 10.1007/s00359-023-01674-2 PMC11226509 37853248

[CR47] Hamanaka Y, Hasebe M, Shiga s (2023) Neural mechanism of circadian clock-based photoperiodism in insects and snails. J Comp Physiol A 210. https://doi.org/10.1007/s00359-023-01662-6 10.1007/s00359-023-01662-6 PMC11226556 37596422

[CR16] Helfrich-Förster C (2023) David S. Saunders: man of insects and photoperiodism (1935–2023). J Comp Physiol A 2010. 10.1007/s00359-023-01665-3 10.1007/s00359-023-01665-3 PMC11226474 37543964

[CR17] Helfrich-Förster C (2024) Erwin Bünning and Wolfgang Engelmann: establishing the involvement of the circadian clock in photoperiodism. J Comp Physiol A 210. 10.1007/s00359-024-01704-7 10.1007/s00359-024-01704-7 PMC11226508 38805044

[CR18] Helm B, Liedvogel M (2024) Avian migration clocks in a changing world. J Comp Physiol A 210. 10.1007/s00359-023-01688-w 10.1007/s00359-023-01688-w PMC11226503 38305877

[CR19] Hidalgo S, Chiu JC (2023) Integration of photoperiodic and temperature cues by the circadian clock to regulate insect seasonal adaptations. J Comp Physiol A 210. 10.1007/s00359-023-01667-1 10.1007/s00359-023-01667-1 PMC11057393 37584703

[CR45] Kaniewska MM, Chvalová D, Dolezel D (2023) Impact of photoperiod and functional clock on male diapause in *cryptochrome *and *pdf *mutants in the linden bug *Pyrrhocoris apterus*. J Comp Physiol A 210. https://doi.org/10.1007/s00359-023-01647-5 10.1007/s00359-023-01647-5 37302092

[CR20] Kramer G (1952). Experiments on bird orientation. IBIS.

[CR21] Kronfeld-Schor N, Bloch G, Schwartz WJ (2013). Animal clocks: when science meets nature. Proc R Soc B.

[CR22] Lankinen P, Kastally C, Hoikkala A (2021). Nanda-Hamner curves show huge latitudinal variation but no circadian components in *Drosophila montana* photoperiodism. J Biol Rhythms.

[CR23] Lees AD (1973). Photoperiodic time measurement in the aphid *Megoura viciae*. J Insect Physiol.

[CR24] Michel S, Kervezee L (2023) One seasonal clock fits all? J Comp Physiol A 210. 10.1007/s00359-023-01680-4 10.1007/s00359-023-01680-4 PMC11226558 37947808

[CR25] Monecke S (2024) Threatened chronotypes: can chronobiology help endangered species? J Comp Physiol A 210. 10.1007/s00359-024-01692-8 10.1007/s00359-024-01692-8 38421416

[CR26] Mouritsen H, Frost BJ (2002). Virtual migration in tethered flying monarch butterflies reveals their orientation mechanisms. Proc Natl Acad Sci USA.

[CR27] Oda GA, Valentinuzzi VS (2023) A clock for all seasons in the subterranean. Comp Physiol A 210. 10.1007/s00359-023-01677-z 10.1007/s00359-023-01677-z 37815602

[CR28] Pittendrigh CS, Daan S (1976). A functional analysis of circadian pacemakers in nocturnal rodents. I. The stability and lability of spontaneous frequency. J Comp Physiol A.

[CR29] Pittendrigh CS, Daan S (1976). A functional analysis of circadian pacemakers in nocturnal rodents: IV. Entrainment: pacemaker as clock. J Comp Physiol A.

[CR30] Pittendrigh CS, Daan S (1976). A functional analysis of circadian pacemakers in nocturnal rodents: V. Pacemaker structure: a clock for all seasons. J Comp Physiol A.

[CR31] Pittendrigh CD, Minis DH (1964). The entrainment of circadian oscillations by light and their role as photoperiodic clocks. Am Nat.

[CR32] Saunders DS (1974). Evidence for ‘dawn’ and ‘dusk’ oscillators in the *Nasonia* photoperiodic clock. J Insect Physiol.

[CR33] Saunders DS (1978). Internal and external coincidence and the apparent diversity of photoperiodic clocks in the insects. J Comp Physiol A.

[CR35] Saunders DS (2023) Time measurement in insect photoperiodism: external and internal coincidence. J Comp Physiol A 210. 10.1007/s00359-023-01648-4 10.1007/s00359-023-01648-4 PMC11226529 37697123

[CR34] Saunders DS, Lewis RD (1987). A damped circadian oscillator model of an insect photoperiodic clock: III. Circadian and hourglass responses. J Theor Biol.

[CR36] Schwartz WJ, Helm B, Gerkema MP (2017). Wild clocks: preface and glossary. Phil Trans R Soc Lond B Biol Sci.

[CR37] Stehlik J, Zavodska R, Shimada K (2008). Photoperiodic induction of diapause requires regulated transcription of timeless in the larval brain of *Chymomyza costata*. J Biol Rhythms.

[CR38] Takeda M, Suzuki T (2022). Circadian and neuroendocrine basis of photoperiodism controlling diapause in insects and mites: a review. Front Physiol.

[CR44] Vaze KM, Helfrich‐Förster C (2016)* Drosophila ezoana* uses an hour‐glass or highly damped circadian clock for measuring night length and inducing diapause. Physiol Entomol 41(4) 378-389. https://doi.org/10.1111/phen.12165 10.1111/phen.12165 PMC5108423 27867253

[CR39] Vaze KM, Manoli G, Helfrich-Förster C (2023) *Drosophila ezoana* uses morning and evening oscillators to adjust its rhythmic activity to different daylengths but only the morning oscillator to measure night length for photoperiodic responses. J Comp Physiol A 210. 10.1007/s00359-023-01646-6 10.1007/s00359-023-01646-6 PMC11226516 37329349

[CR40] Wood S, Loudon A (2014). Clocks for all seasons: unwinding the roles and mechanisms of circadian and interval timers in the hypothalamus and pituitary. J Endocrinol.

[CR41] Yoshii T, Rieger D, Helfrich-Förster C (2012) Two clocks in the brain – an update of the Morning and Evening oscillator model in *Drosophila*. In: In Andries Kalsbeek, Martha Merrow, Till Roenneberg and Russell G. Foster, editors: Progress in Brain Research, Vol. 199, Neurobiology of Circadian Timing. Amsterdam, The Netherlands, pp. 59–82 10.1016/B978-0-444-59427-3.00027-7 22877659

[CR42] Yoshii T, Saito A, Yokosako T (2023) A four-oscillator model of seasonally adapted morning and evening activities in *Drosophila melanogaster*. Comp Physiol A 210. 10.1007/s00359-023-01639-5 10.1007/s00359-023-01639-5 PMC11226490 37217625

[CR43] Zupanc GKH, Homberg U, Helfrich-Förster C, Warrant E, Simmons AM (2024). One hundred years of excellence: the top one hundred authors of the Journal of comparative physiology A. J Comp Physiol A.

